# Evaluating referrals to three urban specialist mental health services for people experiencing homelessness over a 1-year period

**DOI:** 10.1177/00207640251317021

**Published:** 2025-06-28

**Authors:** Margaret Gallagher, Rona Hunt, Eimear Kelly, Jo-Hanna Ivers

**Affiliations:** 1 Department of Public Health and Primary Care, Institute of Population Health, School of Medicine, Trinity College Dublin, Ireland; 2 University College, Dublin, Ireland; 3 The Programme for the Homeless, Usher’s Island, Dublin, Ireland; 4 Department of Psychiatry, The Coombe Hospital, Dublin, Ireland; 5 Department of Psychiatry, The Ashlin Centre, Beaumont Hospital, Dublin, Ireland

**Keywords:** Homelessness, community mental health services, mental illness, psychiatry

## Abstract

**Background:**

There are high numbers of people experiencing homelessness (PEH) in Ireland. PEH have complex needs, and have higher rates of mental disorder than their housed counterparts. To date, there has been limited research conducted on this population, and their specific needs.

**Aims:**

In this study, we explored the demand for the three specialist mental health services for people experiencing homelessness (MHSPEH) in Dublin by evaluating all referrals received over a 1 year period.

**Methods:**

Participants included all persons referred to the three (MHSPEH) over 1 year between 1st July 2022 and 30th June 2023. We examined several key aspects of psychiatric service provision for the population including; population characteristics, psychiatric and Medical History, referral outcomes, alternative pathways and complex health needs.

**Results:**

In total 284 referrals were analysed in the study across the three teams. One third of referrals were accepted overall. Many of the referrals declined did not meet referral criteria, either due to paucity of information, or insufficient evidence of serious mental illness within the referral. Of the referrals accepted, more than half had a previous diagnosis of major mental illness, and 59% were found to be actively psychotic on initial assessment. The majority of those accepted had previous contact with mental health services. One third of the cohort had specific vulnerabilities including: intravenous drug use, international protection applicants and recent release from prison. There were high rates of co-morbid substance and alcohol use in the referrals that were accepted and declined. The majority of referrals were from other psychiatry teams.

**Conclusions:**

There are high rates of mental illness and co-morbid vulnerabilities found in this population. Appropriately addressing the needs of this population will require an integrated, multisystem approach.

## Introduction

Homelessness is a significant social, economic and health issue, resulting in significant costs for the individual, communities and society (Pleace, 2015). It occurs due to the interaction of a variety of dynamic macro and micro circumstances which vary from region to region (Lee et al., 2021). Rates are high internationally, with at least 2 million people experiencing homelessness in OECD countries in 2023 (OECD, 2024). Rates of homelessness have risen steadily in many countries including Scotland, England and Ireland, while other countries like Finland and Norway have seen a steady reduction in numbers experiencing homelessness (O’Sullivan et al., 2023).

People experiencing homelessness (PEH) have higher morbidity, mortality (Aquin et al., 2017; Fazel et al., 2014) and risk of mental disorders (Barry et al., 2024; Nilsson et al., 2024) than their housed counterparts. The standardised mortality ratios of PEH in Ireland are estimated to be 3 to 10 times higher in homeless men and 6 to 10 times higher in homeless women compared with the general population (Ivers et al., 2019). Two thirds are estimated to suffer from mental health disorders (Barry et al., 2024), with rates of psychotic illness being approximately three times higher (3%–42%) in PEH compared to the general population (Fazel et al., 2014). Other common mental health difficulties among PEH include major depression (11.4%), personality disorder (2.2%–71.0%) and substance use disorder (SUD; 4.7%–54.2%; Fazel et al., 2008).

PEH face significant barriers to accessing health care including stigma, discrimination and lack of secure housing (Mejia-Lancheros et al., 2021). This cohort demonstrate lower engagement with primary or preventative healthcare, and typically present at advanced stages of physical and mental illness, both internationally (Fazel et al., 2014) and in Ireland (O’Carroll & Wainwright, 2019). PEH have high rates of co-morbid mental illness and SUD (Fazel et al., 2014), the co-occurrence of which is termed ‘dual diagnosis’ (Torrens et al., 2015).

In many countries, a systemic shift from inpatient to community psychiatric treatment (termed ‘deinstitutionalisation’) led to significant reductions in access to inpatient psychiatric care (Burns & Catty, 2002), with most western countries investing in community, forensic and psychiatric rehabilitation resources to compensate (Chow & Priebe, 2016; Priebe et al., 2008). This did not occur however, in Ireland, where mental health services (MHS), were victim to decades of underfunding, and continue to be allocated less than 6% of the overall healthcare budget (Department of Health, 2023). Experts estimating this allocation would need to be doubled to compensate for this chronic deficit in funding (Griffin, 2023). Consequentially, Ireland has among the poorest access to both general and forensic inpatient care in Europe (Tomlin et al., 2021). PEH seeking access to mental health care in Ireland face several barriers to care, including the fact that MHS are allocated based on a residential address (Johnson & Thornicroft, 1993). PEH are often excluded from accessing care due to not having a fixed address, as CMHT are under significant pressure nationally (Devine & Bergin, 2020). Due to systemic issues, those with dual diagnosis also experience barriers to care and, in some cases, are unable to receive treatment for either disorder (Proudfoot et al., 2019).

There are several, treatment-based models of care which have been shown to improve outcomes for PEH with both mental health difficulties and serious mental illness (SMI), compared to standard case management. Assertive Community Treatment (ACT; considered the gold standard treatment for PEH with SMI), reduces both the severity of psychiatric symptoms and length of homelessness (Coldwell & Bender, 2007). Intensive case management which has been associated with improved engagement and social functioning (Dieterich et al., 2017). Shelter outreach/engagement models have also been found to be effective (Laliberté et al., 2022; Voisard et al., 2021). Unfortunately, there is very limited access to specialist mental health teams for people experiencing homelessness (SMHTPEH) in most areas CMHTs are not resourced to provide assertive outreach to PEH (College of Psychiatrists in Ireland, 2011).

Other effective models of care include ‘Housing first’ (HF), which addresses the housing needs of PEH ‘first’ without prerequisites of sobriety and stable mental health (Tsemberis et al., 2004). HF has been shown to reduce recidivism and hospitalisation (Srebnik et al., 2013). Critical time intervention (CTI) focussing on reducing risk, duration and consequences associated with loss of housing (Herman et al., 2007), and has been shown to reduce rehospitalisation in PEH (Tomita & Herman, 2012). There is very limited provision for Housing First in Ireland at present, with only 647 in HF tenancies in 2022 in Ireland, planning to increase by a further 1,319 by the end of 2026 (Department of Housing, Local Government and Heritage, 2021).

Homelessness increases use of Emergency Care pathways both internationally (Vohra et al., 2022) and in Ireland, where PEH are 20 times more likely to use Emergency Departments (EDs) than their housed counterparts (Ní Cheallaigh et al., 2017). This is particularly significant for those with co-occurring mental disorders, who are significantly more likely to both rely on the ED, and be readmitted to hospital (Lam et al., 2016). ED overcrowding is a global issue which has been shown to result in reduced standards of carer, higher rates of staff burnout and poorer outcomes overall (Savioli et al., 2022). ED overcrowding has been at critical levels in Ireland for many decades, highlighting a chronic strain on healthcare facilities (Cummins et al., 2022; McGowan et al., 2018).

PEH with mental illness have high rates of ED use to access psychiatric care internationally (Kerman et al., 2020; Lam et al., 2016), and in Ireland where in one urban ED, one third of all presentations referred to acute psychiatry were experiencing homelessness (A. Mcloughlin et al., 2020). Such presentations are more likely to occur out of hours (C. McLoughlin et al., 2021), and are associated with high rates of deliberate self-harm and substance use (A. McLoughlin et al., 2021). This trend not only places significant strain on already stretched resources, but points to a larger systemic issue in the accessibility and availability of appropriate MHS. Furthermore, despite overall despite overall admission rates falling steadily in recent decades, the number of PEH requiring psychiatric admission has been increasing (Daly et al., 2019; Guilfoyle et al., 2023).

Increasing numbers of asylum applicants in recent years has likely placed an increased burden on housing resources (O’Sullivan et al., 2023), leading to increased pressure on homeless services (Baptista et al., 2016). Such trends are increasingly prevalent in Ireland in recent years, with many single males in particular resorting to rough sleeping due to inadequate housing supply (EUAA, European Union, 2022), leading to criticism from Irish media and public outcry (Feehan, 2024; Holland et al., 2024). However, in many countries in Europe, asylum applicants utilising homeless services are not counted as homeless for statistical purposes (O’Sullivan et al., 2023), leading to underreporting.

With homelessness steadily rising in the Irish context, and reliance on emergency accommodation increasing by 40% in the last 2 years (Horvat, 2023), this issue is unlikely to resolve without careful planning and appropriate resourcing. Furthermore, COVID-19 has placed additional strain on already overstretched MHS, with many CMHTs operating with staffing levels at approximately 77% of what is recommended by National Policy (O’Connor et al., 2020). Multi-systems interventions are recommended in addressing the needs of individuals PEH with SMI (Shepherd-Banigan et al., 2023). PEH have been identified as a group that may be difficult to monitor, due to their significant mobility, thus the specific needs of the population may not fully be understood (Blackburn et al., 2017). Fazel et al. (2008) recommend the use of service data and local needs surveys to inform service provision when addressing this area of complex need.

## Aims

This study aimed to investigate the demand for specialist homeless services for people experiencing homelessness in the Dublin region and evaluate the clinical characteristics of this population.

This included:

1. Study Participants: What were the ages and demographics of this population?
2. Background: How many had pre-existing diagnoses and contact with mental health services?
3. Appropriateness of referral: Which referrals were accepted?
4. For the referrals that were declined, which services were they redirected to?
5. Co-morbidities: Which co-morbidities were identified?

## Methods

### Location and context

At the time of the study, the centre of the Irish Homeless crisis was the capital city of Dublin, where 69% of those experiencing homelessness resided (18). During the study period, there were three specialist mental health services for people experiencing homelessness (MHSPEH) in Dublin. Teams A and C were longstanding and Team B has commenced operations in early 2022. A fourth MHSPEH, the ‘Inclusion Health Team’ began operating in 2022 but was not included as it was not yet operational at the commencement of the study period.

Each of the three teams had differing referral criteria, resourcing and geographical ‘catchment’ outlined in Table 1. All teams were primarily resourced to manage people experiencing homelessness, with serious mental illness (SMI), requiring assertive outreach and living in a specific geographical region (mainly Dublin Inner City). In order to maximise efficiency, the two established Specialist Mental Health Services for People Experiencing Homelessness (SMHSPEH; Team A and B) were primarily community-based models, with no access to inpatient beds.

**Table 1.** Description of all SMHSPEHs by inclusion and exclusion criteria, catchment area, services offered and resourcing.

Team	Mental health inclusion criteria	Exclusion	Geographical Catchment area	Services offered	Team resourcing
Team A	SMI. Psychotic illness with drug use. Where there was capacity, those with other mental health difficulties, including personality disorder, where there was a high usage of emergency services, or a recent release from prison.	International protection applicants, those under 18 years and those with previous affiliations with south side MHS	Dublin inner city	Assertive outreach, Clinic, Day Hospital, day Centre	Full MDT – Consultant psychiatrist, two psychiatry trainees, one Community Psychiatric Nurse, Day Hospital Nurses, Occupational Therapist, Social worker and psychologist
Team B	SMI only.	International protection applicants, those under 18 years and those with previous affiliations with south side MHS	A geographical area within the north side of Dublin Inner city and two hostels in the Northwest Dublin area	Assertive outreach, outpatient clinic	Partially staffed MDT – one Fulltime psychiatrist, two psychiatry trainees, one Community Psychiatric Nurse and one mental health social worker
Team C	SMI only.	International protection applicants, those under 18 years and those with previous affiliations with north side MHS	Dublin inner city	Assertive outreach, outpatient clinic	Consultant Psychiatrist (0.7 Whole Time Equivalent), one psychiatry trainee, two Community Mental Health Nurses (CMHNs) and occupational therapist and a Senior Social Worker.
Team D	SMI only.	Those with previous affiliations with north side MHS	Dublin inner city	Assertive outreach and links to inclusion medicine	Not yet in operation at the time of the study

Under a SMHSPEH, acceptance required that the referrer agree to take the patient back for inpatient care. For community referrals (eg, General Practitioner), an agreement would be made between the SMHSPEH and the local catchment area service for inpatient admission. Those not meeting these criteria were referred to local Community Mental Health Teams (CMHTs). Local policies required residency within the geographical sector for 3 months prior to CMHT acceptance, though exceptions could be made at the discretion of the Consultant Psychiatrist.

Those who did not meet these criteria were referred to local Community Mental Health Teams (CMHTs). Local policies dictated that a person should be residing within the geographical sector for a 3-month period prior to acceptance by a CMHT. Those with a shorter period of residence could be considered at the discretion of the Consultant Psychiatrist on the team. In Ireland, most CMHTs are not resourced to provide assertive outreach.

At the time of the study, International Protection Applicants (IPAs) were not accepted by MHSPEH, even if residing in homeless accommodation and requiring assertive outreach. Furthermore, those living in LTA (Long Term Accommodation) for PEH were not accepted by MHSPEH. Both fell under the remit of the local CMHT. Referrals were all paper-based, hard-copy referrals, mostly delivered by post.

## Study method

This study evaluated all referrals to the three SMHSPEH based in Dublin between 1st July 2022 and 30th June 2023. The study was based on routine data collected as part of service evaluation.

### Definitions

We defined homelessness using ‘Ethos light’ European Federation of National Organisations Working with the Homeless (19), including those sleeping rough, in emergency accommodation or accommodation for the homeless, those living in institutions whose stay is prolonged due to lack of housing, those in non-conventional dwellings and those living temporarily with friends/family due to lack of housing.

Active psychosis was defined as the presence of delusions, hallucinations and/or thought disorder.

Serious mental illness (SMI) was defined as those with current symptoms or a history of schizophrenia, schizoaffective disorder, bipolar affective disorder and major depression with psychotic symptoms.

emergency accommodation’ ‘supported temporary accommodation’ rough sleeping

### Ethical approval

The research protocol for this study was approved by the TCD Faculty of Health Sciences, Research Ethics Committee, SJH/TUH REC, CH09 REC and SVUH REC. Initially, pseudonymised datasets were collected to identify the total number of individuals across sites. The dataset was then fully anonymised prior to analysis. No individual patient data is presented.

### Participants

Included all persons over the age of 18 years, referred to Specialist Mental Health Teams for the Homeless within the 1-year study period, 1st July 2022 and 30th June 2023.

## Results

### Specialist mental health services for people experiencing homelessness

In total, there were 284 referral episodes (relating to 271 individuals) during the study period. Of these, 33% (94/284) referrals were accepted and 67% (190/284) declined. Of the total referrals, 74% (209/284) identified as male and 26% (75/284) female. Demographic details for each team are described further in Table 3 below.

**Figure 1.**
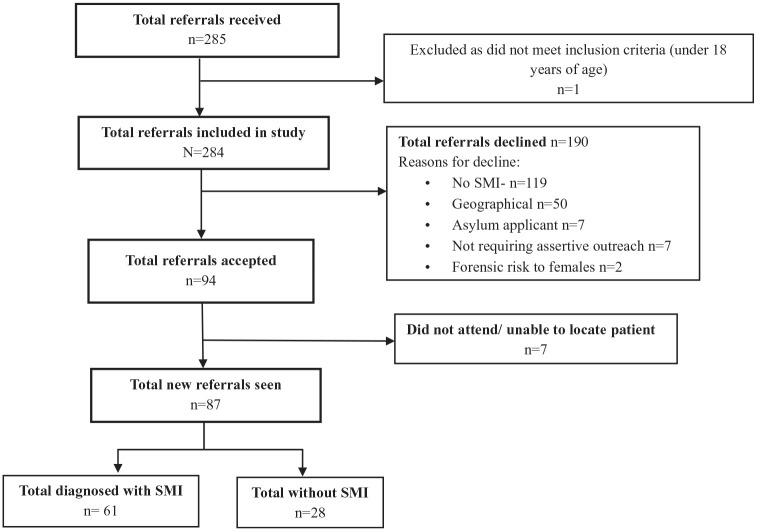
Referrals to review flowchart.

There was considerable variation in the number of referrals and rates of acceptance across the three teams with Team A receiving 149 referrals and accepting 35% (52/149), Team B receiving 20 referrals and accepting 60% (12/20) and team C receiving 115 referrals and accepting 26% (30/115; Figure 1, Table 2).

**Table 2.** Demographics total referrals, age, gender and referrals accepted by team).

Team	Total number	Age	Males	Females	Referrals accepted
		*M*	Number	Number	Total
		Range	%	%	%
Team A	149	39 (18–69)	114 (77%)	35 (23%)	52 (35%)
Team B	20	35 (19–67)	11 (55%)	9 (45%)	12 (60%)
Team C	115	38 (19–62)	85 (74%)	30 (26%)	30 (26%)
Total	284	39 (18–69)	210 (74%)	74 (26%)	94 (33%)

Referrals which were accepted and declined were then analysed separately, as outcomes differed in both groups.

#### Referrals accepted by MHSPEH

##### Demographics

In total, 94 referrals were accepted by the three teams during the study period of which 27% were female and 73% male. The mean age of all accepted referrals was 38 years old. Table 3 describes demographics of all accepted referrals by team.

**Table 3.** Accepted referrals.

Team	*N*=	Male	Female	Age	Previous know contact with CMHT	Currently attending CMHT	Previous dx of SMI	Substance Use	Alcohol use	Number attended for review	Psychotic on assessment
		*N* (%)	*N* (%)	Mean range	*N* (%)	*N* (%)	*N* (%)	*N* (%)	*N* (%)	*N* (%)	*N* (%)
Team A	52	41 (79%)	11 (21%)	39 (21–62)	36 (69%)	10 (19%)	21 (40%)	16 (31%)	6 (12%)	49 (94%)	27 (55%)
Team B	12	6 (50%)	6 (50%)	36 (23–48)	8 (67%)	0 0	5 (42%)	4 (33%)	6 (50%)	9 (75%)	5 (55%)
Team C	30	22 (73%)	8 (27%)	38 (19–61)	25 (83%)	16 (53%)	26 (87%)	16 (53%)	4 (13%)	26 (87%)	23 (88%)
Total	94	69 (73%)	25 (27%)	38 (19–62)	69 (73%)	26 (28%)	52 (55%)	36 (38%)	16 (17%)	84 (89%)	55 (59%)

##### Source of referral

Nearly half (49%) of accepted referrals were from community or hospital-based Psychiatry Teams with a further 12% being referred by Prison Psychiatry Teams. The remaining referrals included 34% from other medical professionals, including GPs, nurses and other doctors and 5% from non-medical referrals including support workers and family members.

##### Presenting complaint, previous diagnosis and previous contact with MHS

Regarding presenting complaint, 79% (74/94) of all referrals accepted by the MHSPEH were for psychotic disorders. Of the 94 accepted referrals, 67% (63/94) were for acute psychotic illness, and 12% (11/94) for chronic psychotic illness which had been stabilised by a but now required transfer of care for ongoing follow-up. Of the remaining 21% (20/94) of referrals not referencing psychosis, 12% (11/94) were for suicidal ideation, 5% (5/94) low mood, 2% (2/94) anxiety, 1% (1/94) PTSD and 1% (1/94) homicidal ideation.

At the time of referral, 28% (26/94) of all accepted referrals were currently attending a CMHT, 70% (66/94) not currently attending a CMHT and the remaining 2% (2/94) unknown. Of the 94 cases, 73% (69/94) had pre-existing contact with MHS, 20% (19/94) had no previous contact with MHS and in 7% (6/94) cases, this was unknown.

In terms of previous diagnosis, 18% (17/94) had no previous diagnosis of mental health difficulties, 13% (12/94) were unknown and the remaining 69% (64/94) had a previous diagnosis recorded on the referral. Of those with a previous diagnosis recorded, 85% (55/65) had SMI including schizophreniform disorders 57% (37/65), other psychotic disorders 17% (11/65) and bipolar disorder 11% (*n* = 7). The remaining 15% included major depressive disorder (*n* = 7), post-traumatic stress disorder (*n* = 1), anxiety disorders (*n* = 1), alcohol use disorder and ADHD (*n* = 1).

Regarding current drug use 40% (38/94), referenced current drug use, 45% (42/94) out-ruled current drug use and in the remaining 15% (14/94) it was unknown. Current alcohol use was identified in 17% (16/94) of cases, out ruled in 60% (56/94) of cases and unknown in 23% (22/94).

##### Other vulnerabilities identified

Of the referrals that were accepted, specific vulnerabilities were identified in 17% (16/94) of individuals. This included a recent release from custody (*n* = 6), active intravenous drug use (*n* = 4), international protection applicants (*n* = 2), recent marital breakdown (*n* = 2) and recent arrival to Ireland from overseas within 1 month (*n* = 2). No individuals were identified as having more than one specific vulnerability.

##### Outcomes

Of the 94 referrals accepted, 65% (61/94) met the criteria for ‘SMI’, 28% (26/94) did not meet the criteria for SMI and the remaining 7% (7/94) did not attend/or were unable to be located. Of those who were seen by the SMHS 82% (71/87) were offered follow-up with the service after their initial appointment, of which seven declined. Of the remainder, 8% (7/87) moved out of the ‘catchment area’ and were referred to their local CMHT, 7% (6/87) were found not to require follow-up with MHS and referred back to their GP, 2% (2/87) was referred for involuntary admission 1% (1/87) were referred to community alcohol and drugs services.

Regarding the period of time experiencing homelessness, in 52% (49/94) of cases this was ‘unknown’, 10% (9/94) were less than 6 months, 12% (11/94) 6 to 12 months, 19% (18/94) 1 to 5 years and in 7% (7/94) 5 to 10 years.

Type of accommodation was not recorded in 10% (9/94) cases. Accommodation type recorded included short-term ‘emergency accommodation’, in 75% (71/94), ‘supported temporary accommodation’ in 10% (9/94) and rough sleeping in 5 (5/94%).

#### Referrals declined by MHSPEH

##### Source of referral and reason for decline

Source of referrals included general psychiatrists 47% (90/190), general practitioners, other medical doctors or nurses 31% (58/190), 14% (26/190) non-medical referrals including key workers, hostel workers and probation. The remaining 8% (16/190) were from forensic psychiatry.

Reasons for the decline included no SMI identified 62% (117/190), 13% (24/190) redirected to another homeless team, 12% (23/190) geographical reasons, 4% (7/190) no felt to be requiring assertive outreach, 4% (7/190) declined due to status as International Protection Applicants, 3% (5/190) of referrals were felt to have insufficient information with the remaining already attending CMHT (*n* = 2), not felt to meet the of homeless (*n* = 2) with the remainder declined on the basis of risk to others (*n* = 2).

##### Presenting complaint, previous contact with MHS and previous diagnosis

With respect to clinical presentation 39% (75/190) referrals made reference to probable active psychosis (*n* = 59) or a known diagnosis of psychotic disorder (*n* = 16), suicidal ideation or deliberate self-harm accounted for 16% (30/190), low mood/depressive disorders for 12% (23/190), anxiety disorders for 8% (15/190) and behavioural changes for 8% (15/190). The remaining 17% included personality disorder (*n* = 7), adjustment disorder (*n* = 7), Post Traumatic Stress Disorder (*n* = 7), eating disorder (*n* = 3), ADHD (*n* = 1) and 7 ‘unknown’.

Previous contact with MHS in Ireland or overseas was recorded in 66% (125/190) cases, no previous contact with MHS in 18% (35/190) and no information was recorded in 15% (30/190). In 45% (85/190) cases there was evidence of current or past psychotic illness.

Of the referrals that were declined, 16% (30/190) with recorded as currently attending a CMHT, 76% (145/190) not currently attending a CMHT and 8% (15/190) were unknown (Table 4).

**Table 4.** Declined referrals.

Team	*N*=	Male	Female	Age	Previous know contact with CMHT	Currently attending CMHT	Previous dx of SMI	Substance use	Alcohol use
		*N* (%)	*N* (%)	Mean range	*N* (%)	*N* (%)	*N* (%)	*N* (%)	*N* (%)
Team A	97	73 (75%)	24 (25%)	38 (20–62)	61 (63%)	17 (18%)	32 (33%)	39 (40%)	18 (19%)
Team B	8	5 (63%)	3 (27%)	34 (19–67)	6 (75%)	1 (13%)	1 (13%)	4 (50%)	1 (13%)
Team C	85	63 (74%)	22 (26%)	40 (18–69)	58 (68%)	12 (14%)	17 (20%)	39 (46%)	34 (40%)
Total	190	141 (74%)	49 (26%)	39 (18–69)	125 (66%)	30 (16%)	50 (26%)	82 (43%)	53 (28%)

The most common previous diagnoses recorded were of psychotic disorders accounting for 28% (53/190) including schizophrenia (*n* = 42), nonspecific psychosis (*n* = 7) and delusional disorder (*n* = 4). Previous diagnosis was unknown in 28% (53/190) of cases and in 15% (29/190) of cases, there was no previous history of mental illness or mental health difficulties.

The remaining 29% included a diagnosis of personality disorder 11% (22/190), anxiety/depression (6%) 11/190, alcohol and substance use disorders 5% (10/190), bipolar disorder 3% (6/190), attention deficit hyperactivity disorder 2% (4/190) and intellectual disability 2% (4/190).

Of the referrals that were declined, 28% (53/190) recorded alcohol use, 27% (52/190) reported no alcohol use and 45% (85/190) had no information regarding alcohol use. With respect to substance use, 43% (82/190) referenced current substance use, 22% (42/190) reported no substance use and 35% (66/190) made no reference to substance use.

##### Other vulnerabilities identified

Specific vulnerabilities were identified in 30% (57/190) of the cohort. The most common vulnerability was a history of intravenous drug use, with 9% (18/190; half of these with active use). A remaining 7% (13/190) were recently released from prison, 6% (12/190) were current International Protection Applicants, 2% (4/190) had intellectual disability, 2% (4/190) had life-threatening medical issues, 1% with physical disability (*n* = 2), 1% identifying as LGBTQI (*n* = 2) and 1% left state care within the previous year (*n* = 2).

##### Outcomes

Of all 190 referrals, half were redirected to other MHS. Of these 38% (*n* = 73) were redirected to the CMHT and 13% (24/190) to other homeless teams. Of the remainder, 18% were declined. These included 20 referrals from other psychiatry teams including CMHT (*n* = 12) and prison psychiatry teams (*n* = 8). In 17 cases, GP referrals were declined, with alternatives suggested in three cases,

Of the remainder, 12 were advised to refer to addiction services, and 2 patient’s whereabouts were unknown.

Period of time homeless was documented in only 31% (59/190) of referrals, with a range of between 2 weeks and 20 years. The type of accommodation was documented in 80% (150/190) of cases including 64% (122/190) residing in Emergency Accommodation (including *n* = 12 IPAs), 4% (7/190) couch surfing, 4% (7/190) LTA, 4% (8/190) rough sleeping, 3% (5/190) STA and 1% (1/190) residing in a car.

##### Accepted versus declined referrals

**Figure 2.**
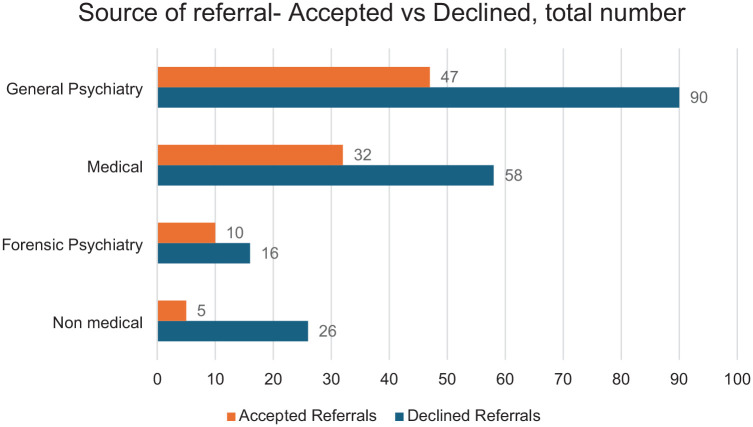
Source of referral-accepted versus declined.

Figure 2 depicts the sources of referral for both the referrals that were accepted and declined. Of the referrals made by general psychiatrists (based in CMHTs and Eds) 67% (90/137) were declined. Of those made by general practitioners, other medical doctors or nurses 64% (58/90) were declined. A remaining 62% (16/26) were declined from forensic (prison based) psychiatry and 84% (5/31) from non-medical sources.

**Figure 3.**
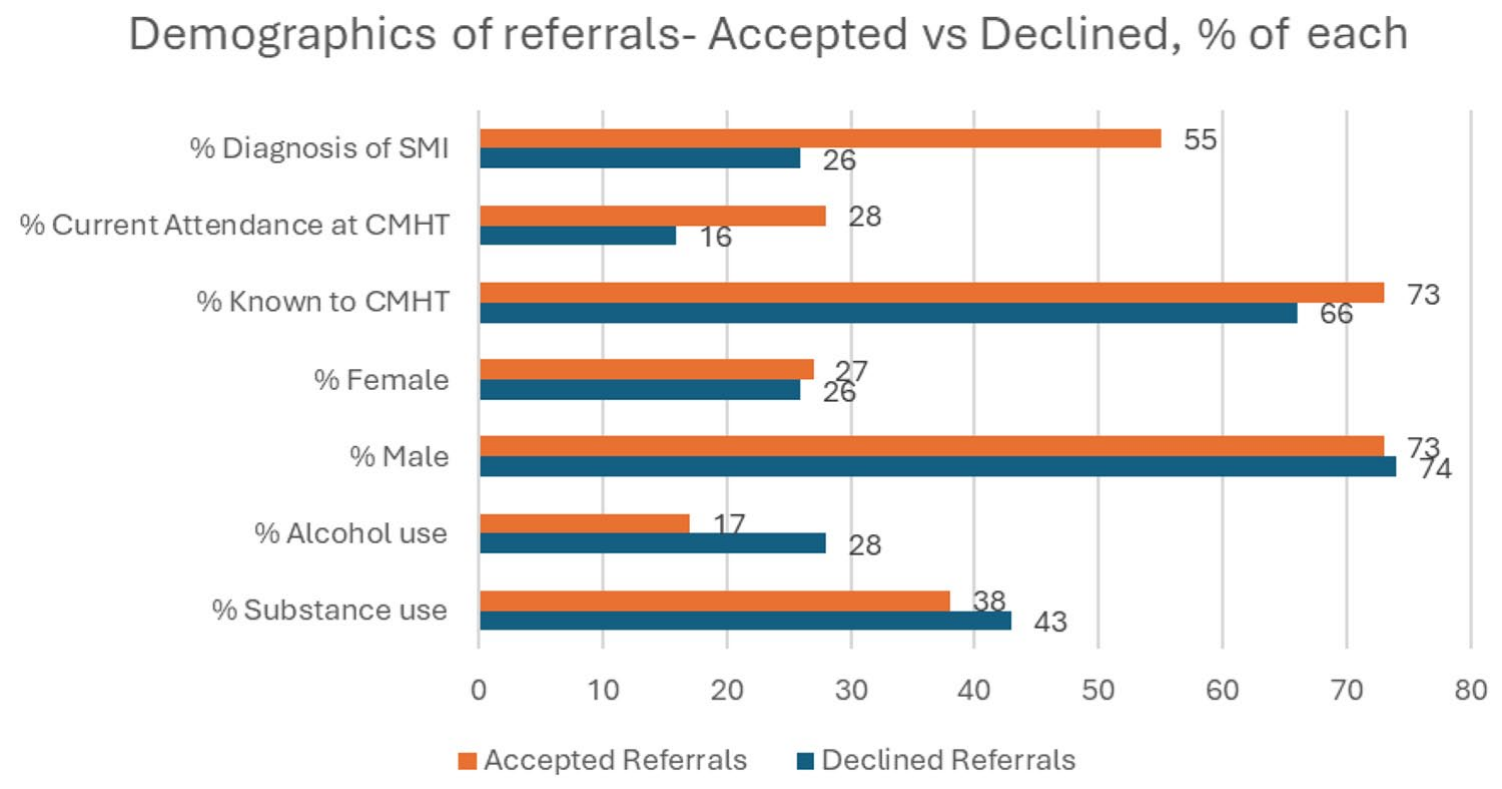
Demographics of referrals-accepted versus declined.

Figure 3 depicts the demographic details of the referrals accepted and declined by %. Higher numbers of those with a pre-existing diagnosis of SMI, or current attendance at CMHT were accepted than declined. Similarly, higher numbers of those previously known to CMHT were accepted. The gender distribution was similar in both groups. While substance use was common in both groups, alcohol use was lower in the accepted group than the declined group.

## Discussion

In this study, we utilised routine service data across three homeless teams to evaluate referrals made to these services. We assessed the referrals which were both accepted and declined by each service and described the demographics of each population.

During the study period, there were no other significant changes to the acceptance criteria within the services, nor was there any major change in the organisation, management or delivery of each of the mental health services.

### Summary of findings

We studied 284 referral episodes to specialist assertive outreach teams for PEH. We have shown that of these, two-thirds of referrals are not accepted by specialist mental health services for the homeless. Referrals evaluated in this study were paper based referrals, with no proforma.

Two thirds of all referrals were declined by SPHSPEH. Many were declined due to not meeting referral criteria (either SMI or geographical), insufficient information or not requiring the specific assertive outreach modality offered by the teams in question. Where referrals were declined, in most cases an alternative pathway was identified by the SMHSPEH. However, in most cases, it was the responsibility of the referrer to arrange and advocate for this onward referral. This creates additional burden on referrers, and likely a delay in accessing appropriate care for the patient. Furthermore, the ‘catchment area’ type model appeared to be a significant disadvantage for those residing in certain areas, with the referral criteria differing across teams.

Of those accepted by MHSPEH, the majority (78%) were suspected to have serious mental illness. In contrast, the majority (62%) of those who were declined were not suspected to have SMI. A high proportion (73%) of those in the accepted group had previous contact with mental health services, with more than half having an established diagnosis of a psychotic illness. Two-thirds of those not accepted had previous contact with MHS, with only 28% of this population having an established diagnosis of psychotic disorders.

Of the referrals that were accepted, most (72%) were not currently under the care of a mental health team, and most (73%) had previous contact with mental health services. Of those not accepted, only 16% were currently attending a CMHT and two-thirds had previous contact with mental health services.

In terms of referral pathway, in terms of those accepted, 61% came from psychiatrists, while 56% of those declined were from psychiatrists. The type of accommodation and length of homelessness was not documented in many cases. High rates of co-morbid alcohol and substance use disorder, as well as specific vulnerabilities were documented in both groups.

There are several studies which may provide suggestions for the appropriate management of referrals. However, these pertain only to UK based CMHTs and their management of GP referrals, as there is a paucity in the literature regarding referrals to CMHTs. According to Chew-Graham et al. (2007), general practitioners and psychiatrists find inflexible acceptance criteria unhelpful when it comes to referring to secondary psychiatric care, and many general practitioners defer seeking input from psychiatry until they have exhausted their own expertise. Flew et al. (2022) suggest good ‘gatekeeping’ practices by psychiatrist can improve efficiency, though warn that significant time can be lost when referrals are declined. The authors also suggest the patient should be kept informed with respect to the decision pertaining to their referral. This was not the practice in our study.

Other authors suggest a liaison type model between primary care psychology and psychiatry to identify those most in need of secondary care services (Hull et al., 2002). Such liaison would require timely access to primary care psychology, which is not the case in Ireland, where primary care psychology is often subject to very lengthy waiting lists (Health Service Executive, 2019).

### Strengths and limitations

This is the first Irish study to examine referrals to specialist mental health teams for PEH. We presented data which was kept for routine service evaluation, allowing for ease of data collection, as well as clinically relevant data. We evaluated all referrals to SMHSPEH in Dublin over a 1-year period, providing a broad and inclusive dataset. Our findings are novel and represent the current clinical activity associated with this population offering important insights into the demand for MHSPEH and outlining the complexity and vulnerabilities associated with this cohort.

This study has a number of limitations. This study relied mostly on routine service data collected from hard-copy paper referrals. While this was an efficient method of data collection, this did not allow quantification or clarification of certain outcomes including further information regarding current symptoms, substance use disorder and other co-morbidities. Additionally, there are significant gaps in documenting the type of accommodation and the length of homelessness, which are critical factors in understanding the context and needs of the homeless population

Local policies dictated acceptance criteria for each homeless team. As outlined in our methodology, this excluded some of those who would be considered to be experiencing homelessness by FEANTSA (19) including those at risk of homelessness, those residing in LTA homeless accommodation and those residing outside of the ‘catchment area’.

As much of the data was based on paper-based GP referrals, some of the fields were ‘unknown’. Furthermore, this study presents data from a limited dataset and does not include inpatient admissions or other community mental health teams are not presented in this study. The findings are therefore not generalisable.

### Future directions

Our findings highlight the significant difficulties that PEH experienced when accessing mental health services in Ireland. Appropriate implementation of ACT requires well-resourced teams with low case numbers (Bond & Drake, 2015). It is clear from our study that the resources are not currently present to meet demand. Many of the referrals declined in the study came from psychiatrists, either community based who were referring as they felt their team was not adequately resourced to meet the person’s needs, or from prison, attempting to arrange timely follow up on release. The introduction of a fourth SMHSPEH is welcomed. However, consensus regarding acceptance criteria and collaboration across the teams is necessary to avoid duplication of work and improve efficiency. It is well established that SUD is common amongst PEH. The timely development of integrated dual diagnosis services as outlined by MacGabhann et al. (2010) is also crucial in meeting the needs if this population.

Referrals in this study were hard copy, paper based. Electronic health records have been shown to improve the quality of information, quality of patient care and provide information for health planning (Häyrinen et al., 2008). Though recommended by the Committee on the Future of Healthcare – Sláintecare (Houses of the Oireachtas, 2017), this has yet to be available in most public mental health services in Ireland. The introduction of an electronic health record would also reduce administrative burden and improve continuity of care for this vulnerable cohort with complex needs. It may also improve collaboration across agencies.

## Conclusion

In summary, the outcomes of this research reveal the stark and pressing difficulties that PEH face when accessing mental health services in Ireland. Despite the proven efficacy of models like ACT and intensive case management, systemic obstacles remain, particularly concerning the availability of services and the adequacy of inpatient resources. Furthermore, the dependence on general practitioners who frequently lack the necessary specialised training and confidence to address such complex psychiatric disorders exacerbates the situation, leaving a population in great need with insufficient support.

This study unequivocally demonstrates that the current mental health system, with its rigid catchment area policies and declining inpatient resources, fails to meet the needs of PEH, leading to increased emergency department presentations and psychiatric admissions. The patchwork of services, influenced heavily by geographical luck rather than needs-based allocation, points to a clear and pressing need for policy overhaul.

It is imperative that we adopt a bold new approach: one that expands evidence-based outreach models across the nation, integrates mental health services with substance use treatment and social care and rethinks the way we allocate mental health services to accommodate the fluid living situations of PEH. Moreover, a substantial increase in inpatient beds and specialised services for dual diagnosis must be prioritised to address the current crisis.
